# Species-specific metabolites mediate host selection and larval recruitment of the symbiotic seastar shrimp

**DOI:** 10.1038/s41598-023-39527-2

**Published:** 2023-08-04

**Authors:** Alexia Lourtie, Igor Eeckhaut, Jérôme Mallefet, Philippe Savarino, Mathilde Isorez, Lisa Mussoi, Hugo Bischoff, Jérôme Delroisse, Laetitia Hédouin, Pascal Gerbaux, Guillaume Caulier

**Affiliations:** 1 Biology of Marine Organisms and Biomimetics Unit, Research Institute for Biosciences, University of Mons-UMONS, 23 Place du Parc, 7000 Mons, Belgium; 2 Marine Biology Laboratory, Earth and Life Institute, University UCLouvain, Croix du sud 3/L7.06.04, 1348 Louvain-la-Neuve, Belgium; 3 Belaza Marine Station (IH.SM-UMONS-ULIEGE), Toliara, Madagascar; 4 Organic Synthesis and Mass Spectrometry Laboratory, Research Institute for Biosciences, University of Mons-UMONS, 23 Place du Parc, 7000 Mons, Belgium; 5 PSL Research University: EPHE-CNRS-UPVD, USR 3278 CRIOBE, BP 1013, 98729 Papetoai, Mo’orea French Polynesia; 6 Laboratoire d’Excellence CORAIL, Mo’orea, French Polynesia

**Keywords:** Behavioural ecology, Biodiversity, Chemical ecology, Natural products

## Abstract

In marine environments, host selection, defining how symbiotic organisms recognize and interact with their hosts, is often mediated by olfactory communication. Although adult symbionts may select their hosts detecting chemosensory cues, no information is available concerning the recruitment of symbiotic larvae which is a crucial step to sustain symbioses over generations. This study investigates the olfactory recognition of seastar hosts by adult *Zenopontonia* *soror* shrimps and the recruitment of their larvae. We examine the semiochemicals that influence host selection using chemical extractions, behavioural experiments in olfactometers, and mass spectrometry analyses. After describing the symbiotic population and the embryonic development of shrimps, our results demonstrate that asterosaponins, which are traditionally considered as chemical defences in seastars, are species-specific and play a role in attracting the symbiotic shrimps. Adult shrimps were found to be attracted only by their original host species *Culcita* *novaeguineae*, while larvae were attracted by different species of seastars. This study provides the first chemical identification of an olfactory cue used by larvae of symbiotic organisms to locate their host for recruitment. These findings highlight the importance of chemical communication in the mediation of symbiotic associations, which has broader significant implications for understanding the ecological dynamics of marine ecosystems.

## Introduction

Symbiotic relationships, defined as close and long-lasting associations between two different species, are ubiquitous throughout the biosphere^1,2^. The impact of a symbiont on the host fitness varies depending on factors such as the type of symbiosis, the developmental stage of the symbiont, or external stressors like food scarcity or environmental changes^3–5^. However, symbionts always drift off a fitness advantage from the symbiotic relationship, often resulting in a strong host dependence^1,6,7^. Co-evolution is frequent in symbiotic associations and leads to intrinsic physiological, morphological, and behavioural adaptations^6–9^. One key factor that sustains symbiotic relationships across generations is the establishment of a specific recognition, allowing the symbiont to selectively associate with its host^10^.

Host recognition is achieved through multimodal communication driven by various stimuli such as visual, acoustic, and chemical signals^11,12^. Chemical sensing is considered as the most common form of communication in marine environments^13–15^. Chemical signals are specific metabolites referred to as ecomones or semiochemicals^13–15^. In interspecific relationships, three categories of ecomones can be distinguished: allomones, kairomones, and synomones. These molecules provide benefits for either the producing organism, the receiving organism, or both, respectively^16^. As the symbiont always receives a benefit from the symbiosis, the molecules involved in host recognition are either kairomones in parasitic or commensal associations or synomones in mutual relationships^13^.

Although chemical signalling plays a crucial role in the establishment of symbiotic relationships^14,15,17–19^, the exact chemical nature of these signals remains largely unidentified in marine organisms. Currently, only four different host recognition semiochemicals have been identified so far in marine environments ^13,20–22^. These structurally and functionally diverse molecules include the amphiphilic triterpenic saponins involved in the association between sea cucumbers and Harlequin crabs^13^ and the hydrophobic amphikuemin acting as a synomone allowing the sea anemone—clown fish symbiosis^20^. Also, two hydrophobic kairomones were recently discovered, the spinochromes produced by sea urchins and recognized by symbiotic shrimps^22^ and anthraquinones that enable recognition between crinoids and pistol shrimps^21^. These cocktails of molecules are chemical signatures that are specific to the hosts, allowing a precise host selection by the symbionts. In comparison with the large number of kairomones that have been identified in terrestrial environment^23^ and given the widespread occurrence of symbioses in marine environments, a multitude of other host recognition kairomones certainly await discovery.

**Figure 1:**
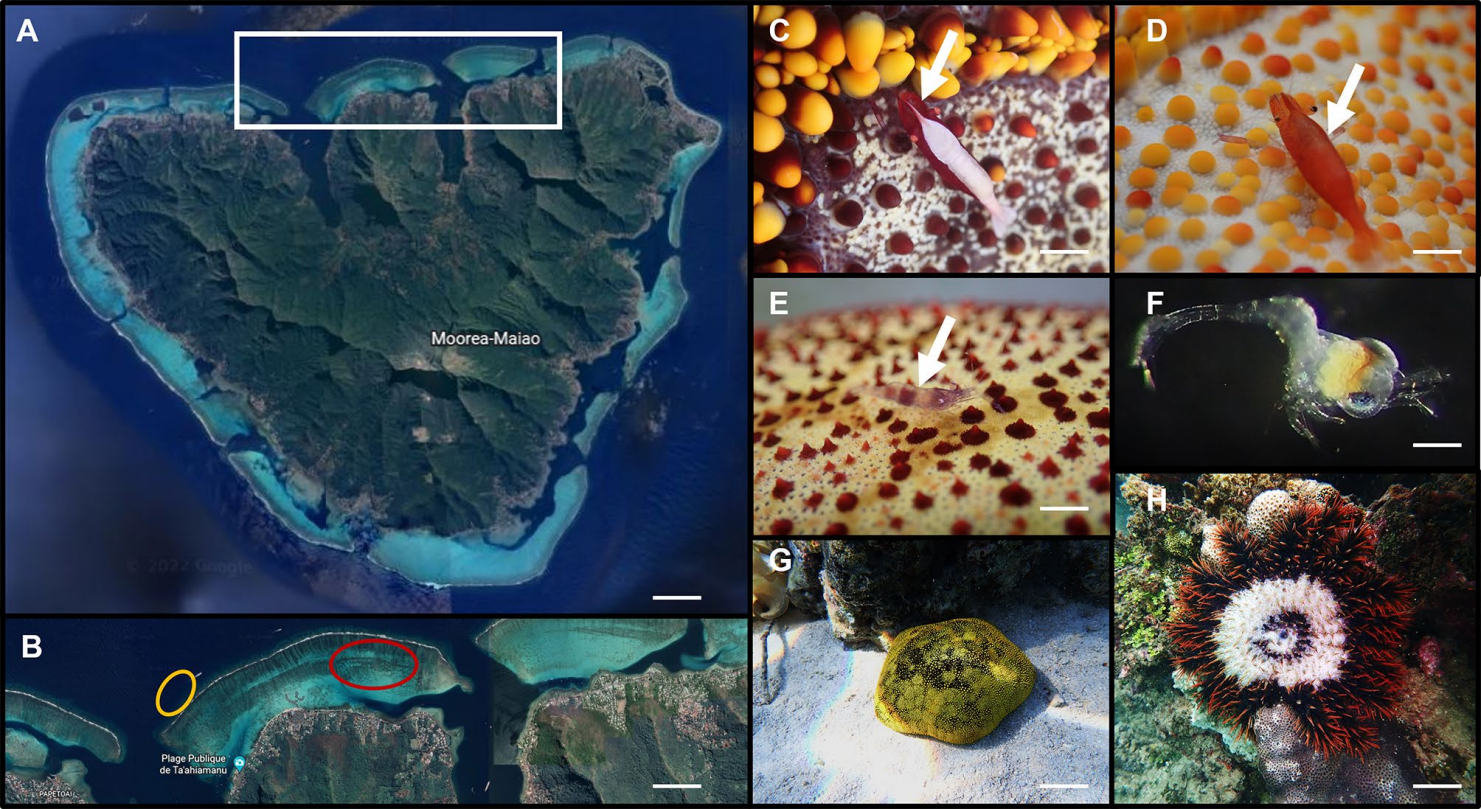
Description of the model organisms investigated and localization: (**A**)  map of Mo’orea Island highlighting the collection area (white rectangle); (**B**) zoom of map (**A**) showing the precise collection site of *Culcita novaeguineae* in red, and the collection area of *Acanthaster planci* in orange. (**C**–**E**) Different morphotypes of the symbiotic shrimps *Zenopontonia soror*, with a white arrow highlighting the symbionts. (**F**) Zoeal larval stage of *Z.* *soror*. (**G**) The host *C. novaeguineae* in its environment and (**H**) *A*. *planci* on a coral colony. (**A**,**B**) Modified from ©Google Earth, version 9.181.0.1. Scale bars represent 0.2 km in (**A**); 1.6 km in (**B**); 0.5 cm in (**C**); 0.6 cm in (**D**; 0.5 cm in (**E**); 100 µm in (**F**); 10 cm in (**G**,**H**).

Considering that most marine symbiotic invertebrates have an indirect life cycle^24^, generating one or several pelagic larval stage(s), the question on how these symbiont larvae can recognize their benthic host to ensure a successful recruitment and settlement remains unanswered. While previous studies have suggested that chemical sensing plays a role in larval settlement^25–29^, this has yet to be demonstrated in the context of marine symbiotic associations. This study aims to address this gap by examining the chemical signals detected by adults and larvae of the symbiotic seastar shrimps *Zenopontonia* (ex. *Periclimenes*) *soror* (Fig. 1C–E) living on the cushion seastar *Culcita* *novaeguineae* (Fig. 1G). *Z.* *soror* is an obligatory symbiont of tropical seastars and is known to be associated with 23 species of asteroids^12^. Asteroid specific steroidal glycosides, also known as asterosaponins^30^, primarily described as metabolites having a role in chemical defence^30^, were here investigated as kairomone candidates. This hypothesis is founded on recent studies proving that echinoderm-specific defensive semiochemicals could also act as pheromones^31^ but also as kairomones in symbioses^13,22^. In the present study, these molecules were tested for the host recognition of adult shrimps, and, for the first time, for the host recognition of their larvae. The scientific strategy involved (1) behavioural tests in two different olfactometer models, (2) saponin extractions from the original host *C.* *novaeguineae*, potential hosts (*Acanthaster* *planci*; *Linckia* *laevigata*)^12,32–36^, and non-host species (*Holothuria* *scabra*; *Asterias* *rubens*; *Echinaster* *sepositus*), and (3) investigation of the diversity of asterosaponins by mass spectrometry analyses.

## Results

### Description of the symbiotic associates

**Figure 2:**
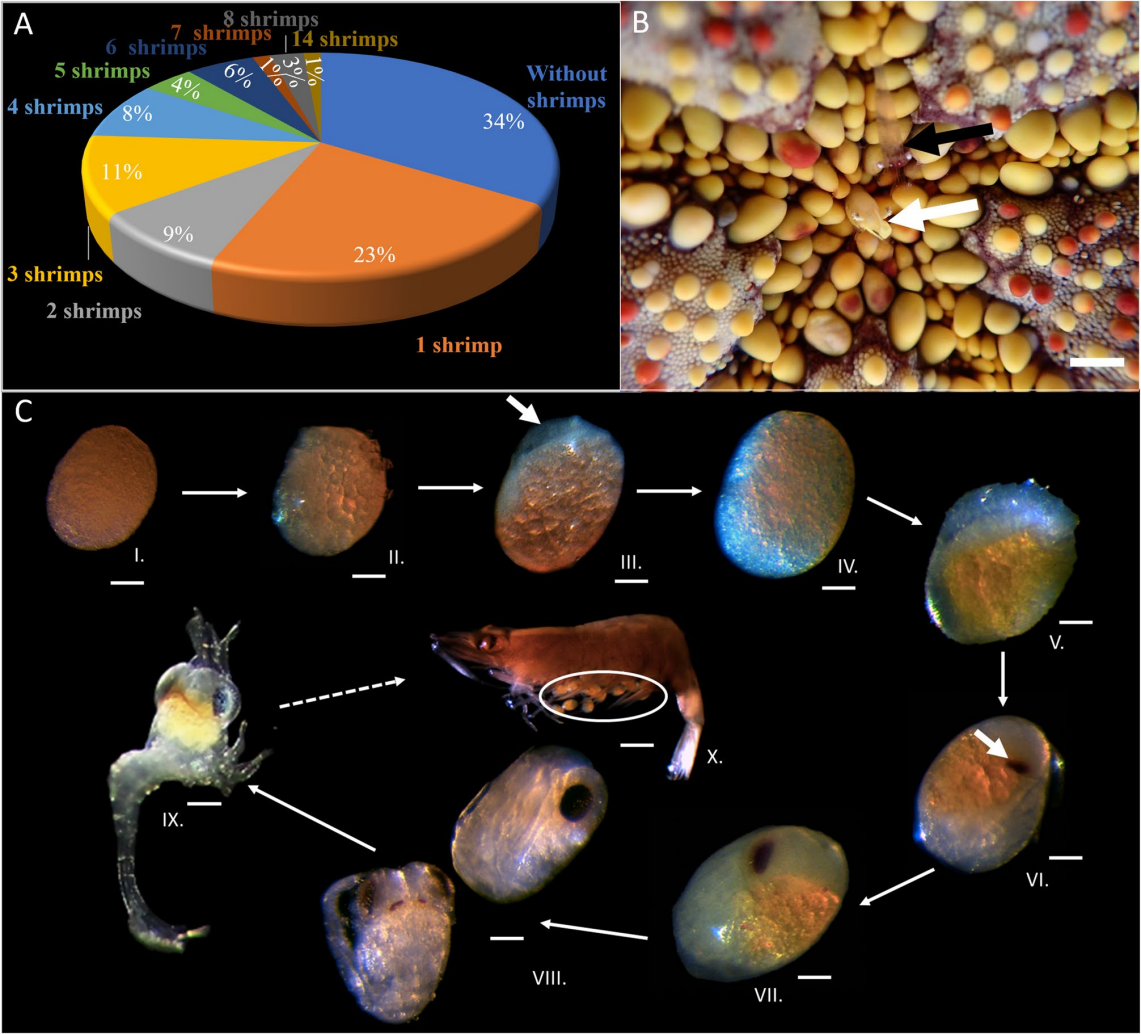
Description of the symbiotic population and the embryonic development of *Zenopontonia soror*: (**A**) occurrence (%) and symbiotic load of seastar shrimps per host individual. (**B**). Two symbiotic *Z. soror* in association with their host *Culcita* *novaeguineae*: one transparent shrimp close to the mouth (black arrow) and one coloured morphotype was observed inside the mouth (white arrow). (**C**)  Developmental cycle of the shrimp *Z.* *soror*. I. Pigmented egg that represents either an unfertilized egg or one of the first development stages. II. Pigmented egg with a large quantity of yolk and larger blastomeres. III. Pigmented egg with a beginning of embryo development (white arrow). IV. Egg with a slight growth of the embryo. V. Pigmented egg with embryo development presenting a discernible antero-posterior axis. VI. Pigmented egg presenting a black vesicle corresponding to the development of an eye (white arrow) and decrease in the quantity of yolk. VII. Advanced developed embryo showing a development of the eye, segmented body and a reduction of the pigmented yolk. VIII. Developed embryo with very little yolk content and developed body and complex eyes displaying ommatidia. IX. First zoeal larval stage. X. Adult gravid *Z.* *soror* with eggs (white circle). Scalebar represents 0.5 cm in (**B**); 140 µm for C I to − C IX and 700 µm in (**C**) X.

A total of 71 *C.* *novaeguineae* seastars, with a diameter varying from 12 to 19 cm (mean ± SD of 14.5 ± 1.3 cm) were collected in the inter-bays of the lagoon of Mo’orea. They were usually hidden under coral patch reefs or rocks during the day and more visible and active during the night in natural and artificial conditions (e.g. climbing the tanks vertical walls each night from the sunset to the sunrise). Sampled cushion stars hosted 152 symbiotic shrimps *Z.* *soror.* The symbiotic occurrence of the association was 69.2%, most shrimps being found externally on the aboral side of their host with 1 to 14 individual(s) per host (Fig. 2A). The mean of the symbiotic load was 2.1 ± 2.6 (mean ± SD) shrimps per seastar. Interestingly, one shrimp individual was observed alive inside the mouth of its host (Fig. 2B). Three colour morphotypes were observed for the symbiotic shrimps: 14.5% (n = 22) were coloured with a dorsal white band (Fig. 1C), 13.2% (n = 20) were fully coloured (Fig. 1D) and 72.4% (n = 110) were transparent (Fig. 1E). Another variability amongst the symbionts concerned their cheliped dimorphism, with 29% (n = 44) of the shrimps presenting an enlarged left claw, 35% (n = 52) having an enlarged right claw and 36% (n = 54) showing no difference between left and right claws. The length of the shrimps ranged from 0.4 to 1.5 cm (mean ± SD of 0.9 ± 0.2 cm) long and 18% (n = 27) were gravid females (Fig. 2CX). Gravid females were the biggest individuals measuring 1.2 ± 0.1 cm long. We also noticed that the occurrence of the symbiosis could dramatically vary inside the lagoon between two micro-populations of cushion seastars that were separated by a navigation channel of 22 m depth in which the flow was stronger, probably limiting the dispersion of the pelagic larvae that could not reach the isolated *C.* *novaeguineae* population. Indeed, no symbionts were found in this isolated population that was not considered in our symbiotic population description. In addition, no symbiotic shrimp was found on the 11 *A.* *planci* (Fig. 1H) observed during the diving collections on the outer reef.

### Description of the developmental cycle

The primary goal of monitoring the shrimp eggs being to obtain larvae that could be tested in a low-flow olfactometer, their developmental stage was regularly monitored and several observations could be made on the development of the embryos. Various developmental stages (from unfertilized oocytes to well-developed embryos) were present at the same time in the population, but for a given female, all eggs presented a homogenous developmental stage A general growth of the egg was observed between the first and the last stage of embryo development, with the most immature eggs (Stage I) measuring 460 ± 28 µm, to the final stage VIII, that exhibited a 639 ± 19 µm diameter (Fig. 2C). In the stages I and II (length = 518 ± 33 µm), only yolk was observed in the eggs through transparency. While the stage I was characterized by numerous “micro-droplets” (probably corresponding to blastomeres) that appeared homogeneous, the next stage comprised bigger “micro-droplets” gathering in one side of the egg (probably corresponding to the growth and migration of the blastomeres toward one pole of the embryo). During stages III (length = 525 ± 36 µm), IV (length = 544 ± 37 µm), and V (length = 555 ± 34 µm), the embryos were visible in the eggs, while a reduction in yolk could be observed. At stage III, an embryo emerged at one pole of the egg. During stage IV, this embryo occupied a larger volume. In stage V, the anterior–posterior axis of the organism could be discerned, as well as the beginning of the segmentation of the locomotor appendages. From stage VI (length = 571 ± 12 µm), the two eyes began to form and presumably contained melanin pigment, developed through stage VII (length = 595 ± 29 µm) and ultimately led to the formation of ommatidia during stage VIII (length = 639 ± 19 µm). Following this stage, the embryo continued to develop, ultimately resulting in the emergence of a zoeal larva. This first stage of zoeal larvae exhibited well-developed appendages, including legs, telson, antennae, and prominent eyes. Measuring 825 ± 35 µm in length, the basal region of the head presented an orange-yellow substance that seemed similar to the yolk. Following larvae developmental stages (e.g. post hatched stages), were not observed in this study.

### Characterization of saponins

**Figure 3:**
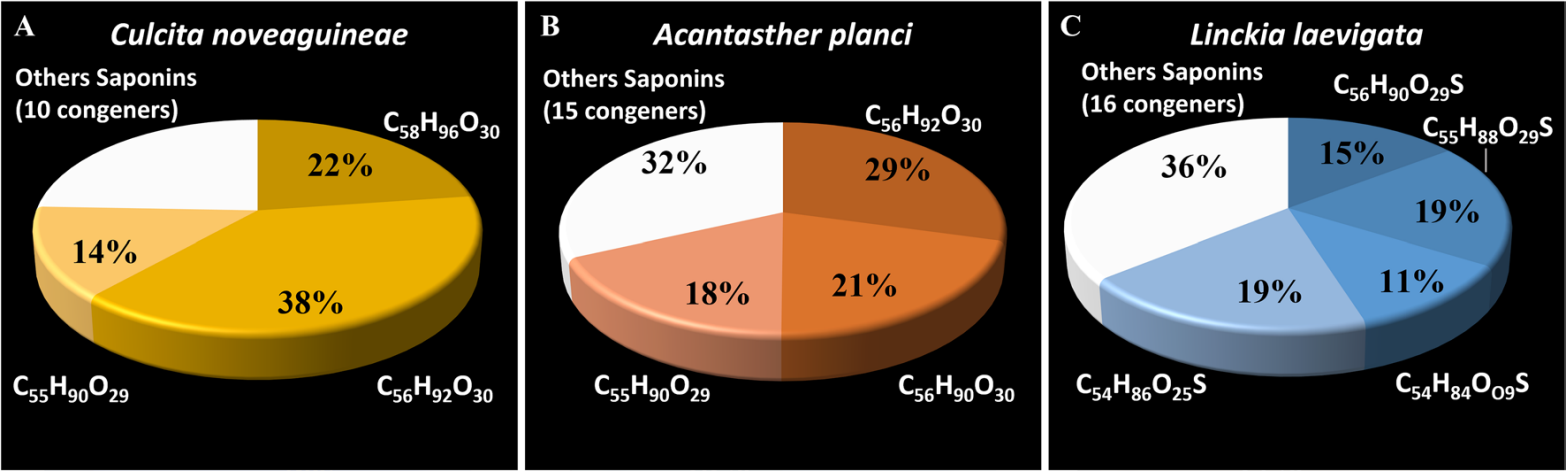
Relative abundances (mol-%) of the asterosaponins of the three potential host seastars: (**A**) *Culcita* *novaeguineae*, (**B**) *Acanthaster* *planci* and (**C**) *Linckia* *laevigata*.

Globally, 51 different saponin molecules were detected in the three potential host species of seastars. Each investigated seastar species presents a specific cocktail of saponins (Table 1; Fig. 3). The saponin congeners were distinguished based on their elemental compositions and LC elution times that are specifically associated with their aglycon/sugar moieties and the presence/absence of a sulphate group (Table 1). The number of detected saponins varied according to the species, with *C.* *novaeguineae*, *A.* *planci* and *L.* *laevigata* presenting 13, 18 and 20 saponin congeners, respectively. Each cocktail was composed of 3 or 4 major congeners that, taken together, represented approximately 75%, 69% and 64% (in relative abundances, mol-%) of the total saponin contents in the natural extract for *C.* *novaeguineae*, *A.* *planci* and *L.* *laevigata,* respectively (Table 1, in bold; Fig. 3). Most of the saponins (n = 37) were species-specific but some saponins (n = 7) were common to both *C.* *novaeguineae* and *A.* *planci* (same molecular formula and retention time). Based on mass spectrometry analyses, we estimated the saponin contents within the natural extracts to be between 92 and 95% (in %-weight).

**Table 1:** Asterosaponins extracted from the three potential seastar hosts, i.e. *Culcita* *novaeguineae*, *Acanthaster* *planci* and *Linckia* *laevigata*.

Saponin	Composition	m/z [M−H]^−^	Δ (ppm)	Retention time (min)	Relative proportion (%)	%-weight in extract (%)
*C.* *novaeguineae*—natural extract—LC–MS(−)—Saponin content (%-weight): 95.7%						
1	C_64_H_106_O_34_	1417.6486	0.1	11.13	2.75	2.63
2*	C_63_H_104_O_34_	1403.6331	0.0	8.23	1.41	1.35
3*	C_62_H_102_O_34_	1389.6173	0.1	8.35	5.56	5.32
4	C_59_H_98_O_30_	1285.6135	4.0	10.30	2.91	2.78
5*	**C**_**58**_**H**_**96**_**O**_**30**_	**1271.5898**	**0.8**	**8.74**	**22.44**	**23.30**
6				11.19	1.91	
7*	C_57_H_94_O_30_	1257.5754	0.2	9.10	1.44	3.83
8				9.24	2.57	
9	**C**_**56**_**H**_**92**_**O**_**30**_	**1243.5591**	**0.3**	**8.42**	**38.39**	**36.74**
10*	C_56_H_92_O_29_	1227.5629	1.4	9.55	3.30	5.94
11*				10.35	2.91	
12*	**C**_**55**_**H**_**90**_**O**_**29**_	**1213.5499**	**0.7**	**8.72**	**13.65**	**13.05**
13	C_42_H_62_O_15_	805.3999	1.5	8.94	0.80	0.77
*A.* *planci*—*Natural* extract—LC–MS(—)—Saponin content (%-weight): 92.4%						
14*	C_63_H_104_O_34_	1403.6331	0.0	8.23	2.15	2.70
15				9.74	0.78	
16*	C_62_H_102_O_34_	1389.6173	0.1	8.35	1.14	1.05
17	C_58_H_96_O_31_	1287.6042	4.0	9.37	0.40	1.81
18				10.30	1.56	
19*	C_58_H_96_O_30_	1271.5898	0.8	8.74	1.26	1.16
20*	C_57_H_94_O_30_	1257.5790	3.0	9.10	4.00	4.09
21				9.87	0.44	
22	**C**_**56**_**H**_**92**_**O**_**30**_	**1243.5599**	**0.3**	**8.84**	**29.30**	**27.07**
23	**C**_**56**_**H**_**90**_**O**_**30**_	**1241.5413**	**2.0**	**8.83**	**1.06**	**21.70**
24				**9.99**	**20.80**	
25				10.80	1.64	
26*	C_56_H_92_O_29_	1227.5629	1.4	9.55	5.14	15.34
27*				10.35	2.43	
28				10.57	9.04	
29*	**C**_**55**_**H**_**90**_**O**_**29**_	**1213.5499**	**0.7**	**8.72**	**18.08**	**16.70**
30	C_54_H_82_O_24_	1113.5160	3.9	10.42	0.30	0.27
31	C_42_H_62_O_15_	805.3999	1.5	9.15	0.50	0.46
*L.* *laevigata*—natural extract—LC–MS(−)—Saponin content (%-weight): 94.1%						
32	C_61_H_98_O_33_S	1391.5740	1.4	7.56	3.93	3.70
33	C_61_H_98_O_32_S	1375.5801	0.7	7.95	1.28	1.20
34	C_60_H_94_O_32_S	1359.5469	2.2	8.91	0.92	0.86
35	C_60_H_94_O_31_S	1343.5530	1.5	8.04	0.62	0.59
36	C_60_H_96_O_31_	1313.6005	1.5	7.52	1.26	1.18
37	C_60_H_96_O_30_	1297.6066	2.3	8.05	2.38	2.24
38	C_59_H_94_O_30_	1283.5860	1.6	7.63	2.73	2.57
39	**C**_**56**_**H**_**90**_**O**_**29**_**S**	**1259.5330**	**0.8**	**8.03**	**14.66**	**13.79**
40	**C**_**55**_**H**_**88**_**O**_**29**_**S**	**1245.5214**	**2.4**	**7.61**	**19.08**	**17.96**
41	C_55_H_86_O_29_S	1243.4978	4.0	8.62	5.33	5.02
42	C_56_H_88_O_28_S	1241.5245	0.8	7.81	4.10	3.86
43	**C**_**54**_**H**_**84**_**O**_**29**_**S**	**1229.4852**	**1.6**	**7.98**	**11.33**	**10.67**
44	C_55_H_82_O_28_S	1223.4727	3.3	7.59	1.57	1.48
45	C_54_H_84_O_28_S	1213.4933	0.8	8.28	3.73	3.51
46	C_56_H_90_O_25_S	1195.5524	1.7	8.98	1.04	0.98
47	**C**_**54**_**H**_**86**_**O**_**25**_**S**	**1167.5232**	**0.0**	**11.23**	**19.13**	**18.00**
48	C_54_H_84_O_25_S	1165.5056	1.7	10.6	1.61	1.52
49	C_54_H_84_O_24_S	1149.5097	2.6	11.23	0.93	0.87
50	C_56_H_86_O_23_	1127.5603	0.9	11.23	1.72	1.62
51	C_55_H_84_O_23_	1113.5447	0.9	8.03	2.65	2.50

Saponin contents (%-weight) were determined relative to the internal standard, hederacoside C (Sigma-Aldrich, Belgium). Major congeners are in bold. Common congeners are highlighted with an asterisk*.

### Behavioural olfactometry analyses on adult *Z.soror*

During the negative control with both aquaria of the olfactometer exclusively filled with non-conditioned seawater, 90% (n = 18/20) of shrimps remained in the entry of the olfactometer and did not show any positive chemotaxis (Table 2; Video SI2). Therefore, *Z.* *soror* individuals did not elicit attractive rheological or chemically-mediated behaviour when testing non-conditioned seawater flows. When an aquarium was conditioned with the initial host, *C.* *novaeguineae* or its extracted asterosaponins, the shrimps usually displayed grooming and flicking behaviours with their A1 and A2 antennae and pereiopods, that are associated to chemoreception patterns^13^. Chemoreception led to the successful detection and orientation of more than 82% (n = 33/40) of the shrimps (Video SI1), which confirms that *Z.* *soror* can detect semiochemicals produced by their initial host but also that asterosaponins play the role of kairomones allowing host selection.

**Table 2:** Results of behavioural experiments using the Y-tube olfactometer on adult *Zenopontonia soror*.

Aquarium A VS aquarium B	Results				Statistics	
	Trials	Null	A	B	Motion behaviour	Orientation behaviour
Negative control						
Seawater (A) VS seawater (B)	20	18	1	1	–	1
Water conditioned by the initial host						
***Culcita*** ***novaeguineae*** **(A)** **VS** **seawater** **(B)**	40	4	**33**	3	**1.8** **×** **10**^**–10**^	**2.3** **×** **10**^**–7**^
Water conditioned by asterosaponins extracted from potential host asteroid species						
*C.* *novaeguineae* extract (A) VS seawater (B)	40	4	**33**	3	**1.8** **×** **10**^**–10**^	**2.3** **×** **10**^**–7**^
*Acanthaster* *planci* extract (A) VS seawater (B)	20	13	6	1	0.1	0.1
***Linckia*** ***laevigata*** **extract** **(A)** **VS** **seawater** **(B)**	20	15	5	0	0.4	**6** **×** **10**^**–2**^
Water conditioned by saponins from non-host echinoderm species						
*Asterias* *rubens* extract (A) VS seawater (B)	20	18	1	1	1	1
*Echinaster* *sepositus* extract (A) VS seawater (B)	20	18	1	1	1	1
***Holothuria*** ***scabra*** **extract** **(A)** **VS** **seawater** **(B)**	20	18	1	1	1	1

Eight different tests were performed on a minimum of 20 different adult shrimps. Results show the number of trials, spread out among the shrimps that remained stationary in the beginning of the impaired branch (Null), those that orientated adequately towards the cues source (A) and those of orientated towards the control aquarium (B). The P values of the chi^2^ test and the binomial test are respectively found in motion and orientation behaviour columns. Significant results (P value, 0.01) are underlined and bolded**.**

Asterosaponins extracted from the potential host crown of thorns seastar *A.* *planci* and the azure seastar *L.* *laevigata,* elicited chemosensation behaviours but were not attractive to the *Z.* *soror* with 65% (n = 13/20) and 75% (n = 15/20) of the tested shrimps remaining at the origin of the Y-tube olfactometer and displaying no positive chemotaxis. Finally, saponins extracted from the two non-host seastar species (*A.* *rubens* and *E.* *sepositus*) and the sea cucumber *H.* *scabra* revealed similar results as the negative control and were thus considered non-attractive for *Z.* *soror*.

### Behavioural low-flow olfactometry analyses on larvae *Z.soror*

The low-flow Y-tube device is adapted to test larval chemotaxis (Fig. 6). Results are summarized in Table 3. When performing the negative control (i.e. both aquaria filled with non-conditioned seawater), the larvae remained quite inactive and were transported passively into the Y-tube with the current generated by the peristaltic pump. The mean (± SD) drifting time of the larvae in the system was 64.3 ± 49.4 s. No detection/orientation behaviour was observed (12 null on 20 larvae). Indeed, larvae almost never moved actively in the device and when they did drift off to the right or the left part with an equivalent percentage (50%). When one of the aquarium was filled with water conditioned by the host species, larvae were more active and swam periodically and remained for a significatively longer time in the monitoring zone (ANOVA, F(4, 175) = 7.1, P value = 2.7 × 10^–5^). The larvae could strongly orientate themselves toward the host side of the Y-tube (33 larvae oriented adequately on 40). Those results confirm the validity of the low-flow olfactometer device that can be used to identify larvae chemotaxis. Interestingly, the zoeal larvae displayed similar orientation results with water conditioned with the saponins extracted from the two potential host species *C.* *novaeguineae* and *A.* *planci*. However, when we performed a test to simultaneously compare the cues originating from the two potential host species, the larvae oriented themselves significatively towards *C.* *novaeguineae*, indicating a preference for this cue compared to the one of *A.* *planci*.

**Table 3:** Results of behavioural experiments using the olfactometer on larvae of *Zenopontonia soror*.

Aquarium A VS aquarium B	Results				Statistics	
	Trials	Null	A	B	Motion behaviour	Orientation behaviour
Negative control						
Seawater (A) VS seawater (B)	20	12	4	4	–	1
Water conditioned by the initial host						
***Culcita*** ***novaeguineae*** **(A)** **VS** **seawater** **(B)**	40	4	**33**	3	**1.3** **×** **10–**^**4**^	**2.3** **×** **10**^**–7**^
Water conditioned by asterosaponins extracted from potential host asteroids						
*C.* *novaeguineae* extract (A) VS seawater (B)	40	5	**33**	2	**3.9** **×** **10**^**–4**^	**3.7** **×** **10**^**–8**^
*Acanthaster* *planci* extract (A) VS seawater (B)	40	6	**32**	2	**1** **×** **10**^**–3**^	**6.4** **×** **10**^**–8**^
Comparison of two waters conditioned by asteroids saponins extracts						
*C.* *novaeguineae* extract (A) VS *A.* *planci* extract (B)	40	8	**29**	**3**	**4.5** **×** **10**^**–3**^	**2.6** **×** **10**^**–5**^

Five different tests were performed on a minimum of 20 different larvae. Three individuals of *Culcita novaeguineae* were used to prepare conditioned water and, for each saponins extract, at least 3 individuals for each species were used. Results show the number of trials, spread out among the shrimps that did not converge significatively toward the device (Null), those that orientated adequately toward the cues from aquarium A (A) and those that orientated toward the cues coming from the aquarium B (B). The P value of the chi^2^ test and the binomial test are respectively found in motion and orientation behaviour columns. Significant results (P value, 0.01) are underlined and in bold.

**Figure 4:**
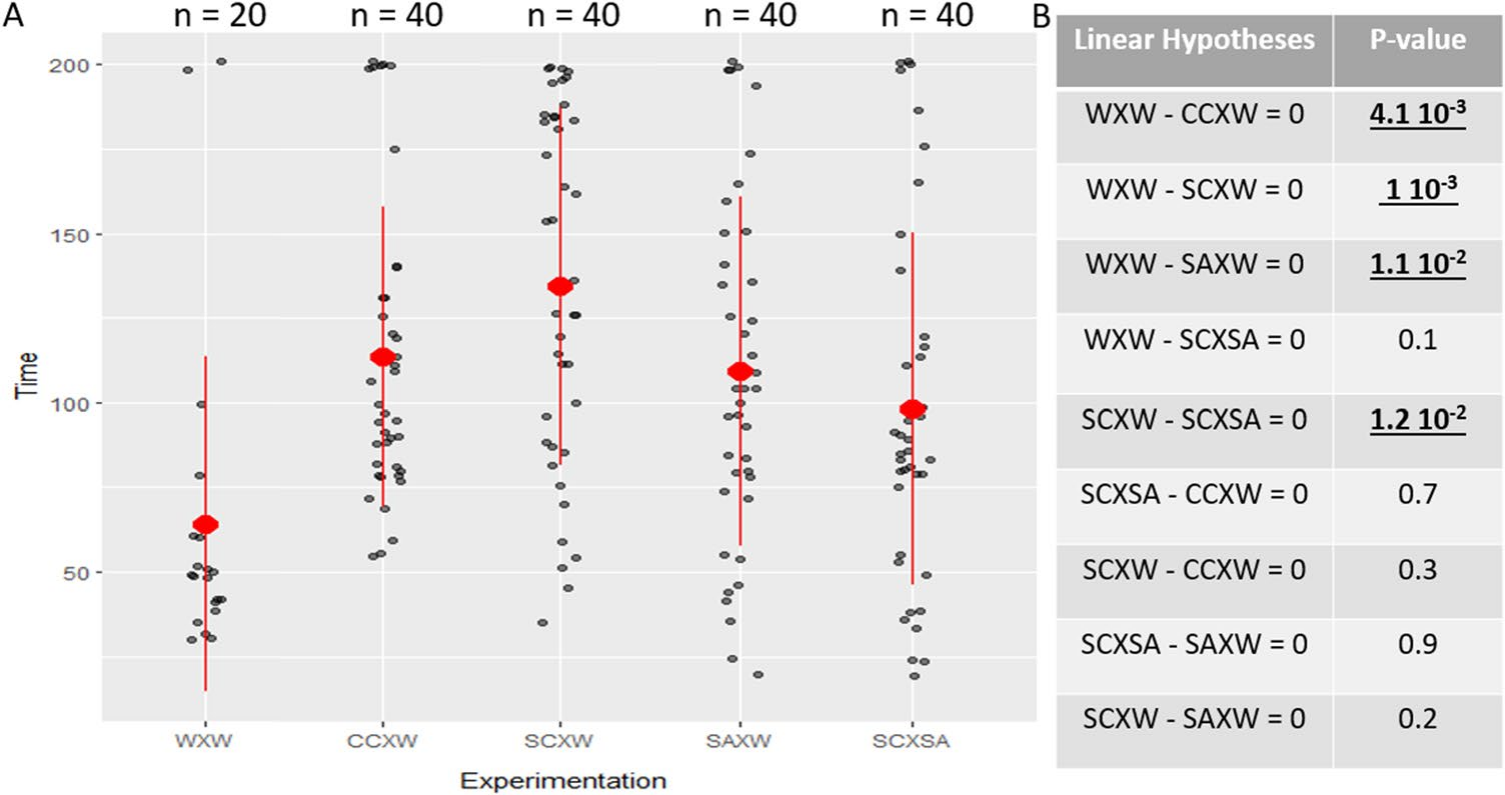
Drifting time of *Zenopontonia* *soror* larvae test under different chemical cues: (**A**) *Z.* *soror* larvae drifting time (s) inside the olfactometer device during the different experimentations. For each experiment, the red spot corresponds to the meantime values and the red line represents the standard deviation. The grey dots represent the measures of the larvae drifting time, with small random horizontal shifts to visually separate them. The number n above each box represents the number of larvae observed in each experimentation. All experiments are abbreviated as the following (i) WXW: seawater VS seawater (negative control); (ii) CCXW: C. novaeguineae conditioned water VS seawater; (iii) SCXW: Saponins from C. novaeguineae VS seawater; (iv) SAXW: Saponins from *A.* *planci* VS seawater. (**B**) Results of the multiple comparisons of mean (Tukey) test. Significant results (P value, 0.01) are underlined and bolded.

The homoscedasticity (Bartlett Test, K^2^ = 1.5, df = 4, P value = 0.8) and the distribution of the residuals were verified using a quantile–quantile plot (Fig. SI-1) and showed that they did not differ significantly from a normal distribution. Multiple comparisons of means (Tukey HSD) demonstrated (Fig. 4) that larvae had a shorter drifting time in the device in the negative control test (seawater VS seawater) than when they are confronted with the cues of one of their hosts [(linear hypotheses = *C.* *novaeguineae* conditioned water VS seawater–seawater VS seawater = 0, t = − 0.4, P value = 4 × 10^–3^); (linear hypotheses = *C.* *novaeguineae* extract VS seawater—seawater VS seawater = 0, t = − 5.1, P value = 1 × 10^–3^); (linear hypotheses = *A.* *planci* extract VS seawater–seawater VS seawater = 0, t = − 3.3, P value = 1 × 10^–2^)]. When both host extracts were injected together, larvae stayed slightly more time in the device in comparison to the negative controls but the mean times are not significantly different (linear hypotheses = *C.* *novaeguineae* extract VS *A.* *planci* extract—seawater VS seawater = 0, t = − 2.5, P value = 0.1). However, a significant mean time difference is observed between the test where both extracts are tested together and the test with only the asterosaponins of *C.* *novaeguineae* are injected, with the larvae staying more time in the second one (linear hypotheses = *C.* *novaeguineae* extract VS seawater—*C.* *novaeguineae* extract VS *A.* *planci* extract = 0, t = 3.2, P value = 1 × 10^–2^).

## Discussion

### Notes on the symbiosis between *Z.soror* and *C.novaeguineae* in Mo’orea

*Zenopontonia* *soror* is an obligate associate that inhabits at least 23 different asteroid host species, making it a generalist symbiont at the species level and a specific symbiont at the Asteroidea class. In the northern Mo’orea lagoon, more than two third of *C.* *novaeguineae* hosted seastar shrimp individual(s), with a maximum of 14 shrimps coexisting on a same host. To date, the highest occurrence ever recorded reached up to 53 individuals of *Z.* *soror* on one *C.* *novaeguineae* ^12,36^. Even if previous studies reported the association between *Z.* *soror* and *C.* *novaeguineae* in Tuamotu islands and Society islands^38,39^ and even describe partially the host preference and food habit of the symbiont^32,34^, this study is the first to provide an exhaustive description of the symbiotic population in French Polynesia. The crown-of-thorns *A.* *planci* monitored in this study did not host any symbiont but were only found on the external reef and not in the lagoon of the North of Mo’orea at the time of the field missions (Fig. 1B). This corallivorous species may be abundant in the waters of Mo’orea including in the lagoons of French Polynesia in general^40–42^. *L.* *laevigata* and *L.* *multiflora* may also be natural hosts in this area^32,43^ but were not observed. These four species are known to coexist in some other geographic areas, such as, for example, the Nhatrang Bay in Vietnam where the symbiotic shrimp was also reported^44^. Additionally, we report, for the first time, that *Z.* *soror*, referred exclusively as an ectosymbiont^12,32–36^, may enter the buccal cavity of its host (Fig. 2B) and could be, under certain conditions (e.g. predation stress), a temporary endosymbiont. As no study has ever investigated specifically the buccal cavity of *C.* *novaeguineae* to monitor the presence of symbionts, the occurrence and symbiotic load of *Z.* *soror* described in literature^44,45^ may be biased as we know that some symbiotic crustaceans associated with echinoderms frequently exhibit an endosymbiotic lifestyle (e.g. the Harlequin crab *L.* *orbicularis*^46^).

We partially described the embryonic development of *Z.* *soror* providing further insights into the life cycle of this species. Similar to non-symbiotic palaemonid shrimps, the development of *Z.* *soror* is complex and involves multiple stages, from fertilization to hatching^47–50^; during which the embryos undergo a series of morphological changes. Each stage is characterized by specific timing and the modification of the size, shape, and functionality of the developing organs and structures^47,48^. The embryonic description complements precedent study describing the larva of *Z.* *soror*^51^. The post hatched zoeal stages have never been observed before. However, in Palaemonidae, the free-swimming larvae may go through 9–13 zoeal stages before reaching the juvenile stage^49^. Traditionally studied for commercial palaemonid species^47–49^, understanding the embryonic development of symbiotic organisms is also important for the management and conservation of symbiotic marine biodiversity.

### Host selection of the seastar shrimp through chemical sensing

**Figure 5:**
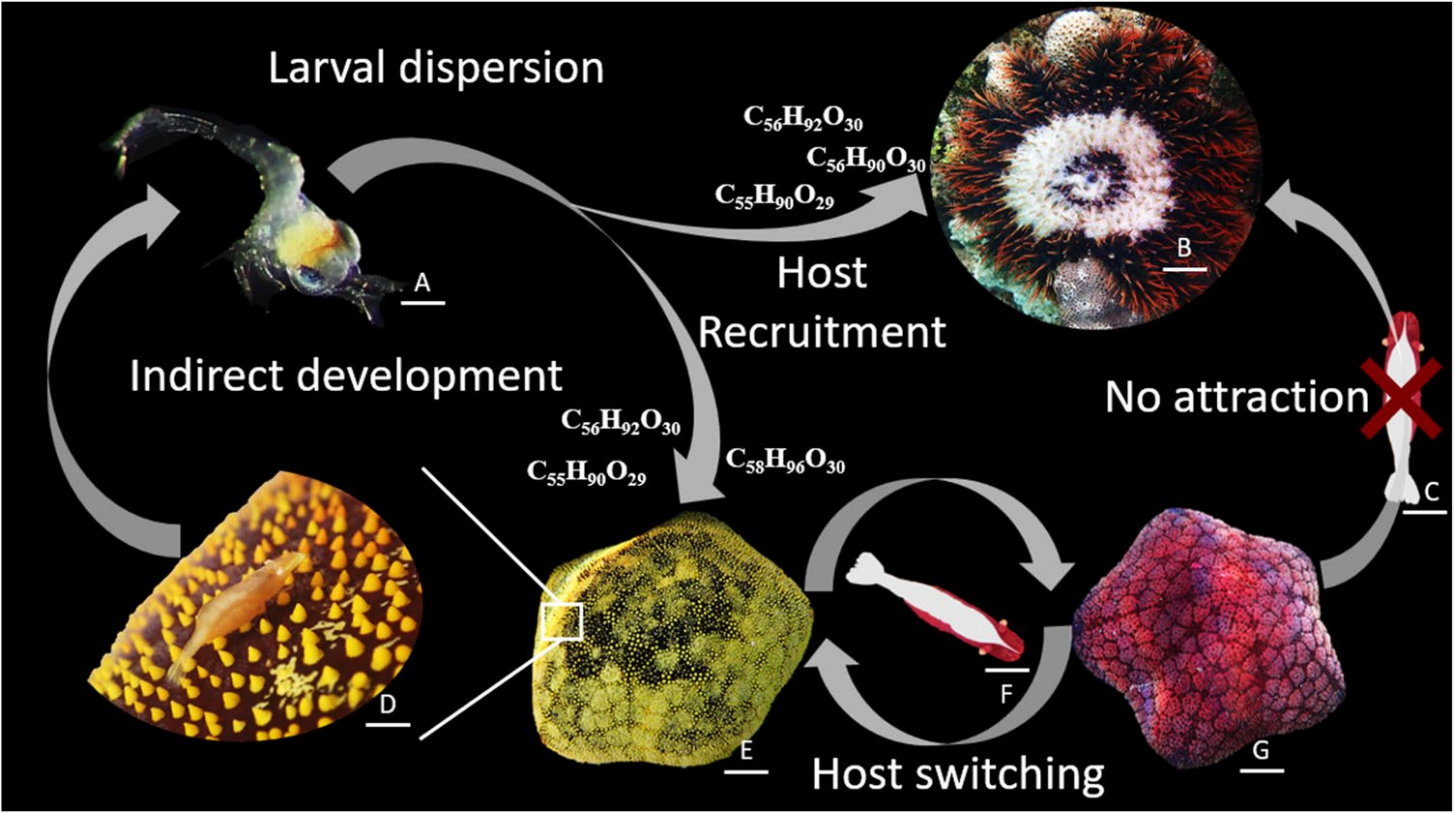
Summary diagram of the mechanisms involved in host recognition, host switch and larval settlements in the symbiotic association between *Culcita* *novaeguineae* and *Zenopontonia* *soror*. Scale bars represent 110 µm in A; 3,6 cm in B; 250 µm in C, D and F; 2 cm in E and G.

Behavioural experiments in olfactometers with adults *Z.* *soror* confirm that host selection of the symbiont is mediated though chemical sensing, as already shown in recent literature^12,34^. Antokhina and Britayev showed that *Z.* *soror* recognizes its original host species through chemical cues, but not the other host species present in the environment^12^. Our innovative low-flow Y-tube olfactometer results prove that seastar chemical cues have an attractive effect on the larvae, eliciting a positive chemotaxis and enabling pelagic larvae to selectively recruit on their benthic hosts before metamorphosis. This ensures the sustainability of the symbiotic association across generations (Fig. 5) and could also explain why molecular genetic study have found that different *Z.* *soror* populations associated with different asteroid species are genetically homogeneous^52^. *Z.* *soror* exhibits a remarkable ability to form associations with diverse seastar hosts^13,34–38^, thereby accessing a wide range of habitats and promoting gene flow among populations. Essentially, the *Z.* *soror* larvae can find suitable habitats in tropics and drift with the water current if any of the host species is present.

For the first time, the study reveals that these cocktails of asterosaponins act as chemical cues (i.e. kairomones) allowing host selection by both adults and larvae of *Z.* *soror*. Asterosaponins from *A.* *planci* have previously been suggested to serve as a pheromonal recruitment factor allowing (1) its larvae to perform their metamorphosis in areas where other conspecifics are present and (2) the aggregation of adult crown of thorns seastars^53^. In the present study, we compared the asterosaponin contents from different host and non-host species^30,54,55^ (Table 1) and found that each seastar species produces a unique mixture of asterosaponins. This specificity explains the differences exhibited by *Z.* *soror* adults and larvae when exposed to water conditioned by saponins extracted from different potential host species. Indeed, the *Z.* *soror* adults were only attracted by their original host species *C.* *novaeguineae* saponins, on which they were exclusively collected. They were not attracted by the saponins extracted from 5 other echinoderms, two potential host species where *Z.* *soror* adults have already been recorded in literature (*A.* *planci* and *L.* *laevigata*) and 3 non-host species (*A.* *rubens,* *E.* *sepositus* and *H.* *scabra*). It is not surprising that *Z.* *soror* adults are not attracted by non-host species, but the fact that saponins from *A.* *planci* do not elicit a positive chemotaxis on the adults but do attract their larvae can be explained by a phenomenon known as symbiotic host imprinting. Host imprinting explains how a symbiont may co-adapt its chemoreception capacities to recognize the specific host species on which it is found^12,56^. It is well established that host imprinting is a common phenomenon among decapods, for host recognition, habitat preference or food habit for example^12,21,56,57^. At the opposite, Caulier et al*.*^13^ showed that adults of tropical Harlequin crabs *L.* *orbicularis* positively recognize the saponins from *Holothuria* *forskali* (a temperate sea cucumber where they never occur) suggesting that imprinting is not a general rule for all decapods. As soon as they are capable of swimming efficiently*,* *Z.* *soror* larvae are certainly transported by natural seawater currents to different places in the benthos. If, by chance, they contact or are close to a potential host, our experiments suggest that their swimming activity will allow them to stay in contact with that host. Probably taking advantage of the surface of this host, they could stay there long enough to metamorphose. Indeed, we demonstrated that they can detect the presence of different species of potential hosts in their environment and to orient themselves towards them. Then, adults *Z.* *soror* will be imprinted by the original host species, specializing its chemotaxis capacities and being no more attracted by asterosaponins produced by other potential hosts present in the environment.

Our results confirm previous findings that each species of holothuroids and asteroids produces a unique mixture of different saponin congeners, resulting in a distinct cocktail of molecules that can be considered as a chemical signature^30,54,55,58,59^. These molecules can have a role in both pheromonal communication^31^ and host recognition phenomenon^13^. Some asteroid species exclusively produce sulphated saponins while others only produce non-sulphated saponins, and some produce both^59–61^. Moreover, saponins are known to play a crucial role in chemical defence in marine environments and have strong ichthyotoxic properties that effectively deter potential predators^62^. Therefore, symbiotic fauna must evolve mechanisms to counteract the harmful effects of asterosaponins. Furthermore, they may also leverage their host chemical defence mechanisms to protect themselves from their own predators. In the present study, we found that *C.* *novaeguineae* and *A.* *planci* only produce non-sulphated saponins, while *L.* *laevigata* produces both sulphated and non-sulphated asterosaponins. Interestingly, previous studies have shown differences in saponin congener profiles within populations of the same species, which may be explained by the geographical distance between the populations. For example, *C.* *novaeguineae* has been previously studied in South China^63–67^, Vietnam^61^, Japan^68^ and Seychelles^69^. Similar analyses have also been conducted for *A.* *planci* in Japan^70^ and Vietnam^58,59^. Therefore, it would be interesting to compare the potential host attractiveness of asteroids from different parts of the world to their larvae and adult symbionts in future studies. The differences in saponin mixtures among different populations of the same species may also impact intraspecific recognition and aggregation, underlining a potential emergence of a speciation event.

## Conclusion

In this study, we investigated the recruitment of symbiont larvae through chemical sensing. We have shown that the host chemical recognition is not limited to adult symbionts, but also occurs in symbiont larvae. Adults shrimps become imprinted by their initial host species and are no longer attracted to asterosaponins produced by other potential hosts. These results shed light on the mystery of the symbiont larval recruitment and provide valuable information on how these symbionts complete their life cycle.

## Method

Two scientific expeditions were conducted during July and August 2021 and 2022 at the Centre for Island Research and Environmental Observatory (CRIOBE) located in Mo’orea, French Polynesia. Sample collection, larval rearing, behavioural experiments, and chemical extractions were performed on site. Chemical purification and mass spectrometry analyses were performed at the University of Mons (Belgium).

### Sampling, organism maintenance, larval rearing

*Zenopontonia* *soror* (Nobili, 1904) and its most frequent host in the area, *Culcita* *novaeguineae* Müller & Troschel, 1842, were hand-collected using free diving techniques in depths ranging from 1 to 5 m on the northern coast of Mo’orea, located between the Opunohu and Cook Bays (17° 28′ 50″ S; 149° 50′ 00″ W; Fig. 1A,B). The collected seastars were carefully stored in 3 L-hermetic plastic bags filled with seawater to prevent the loss of symbiotic shrimps. Upon arrival in the laboratory, the symbionts were inventoried, their length carefully measured using graph paper, their number recorded and their morphotype determined (Fig. 1C–E). Each shrimp individual investigated in this study originated from a cushion seastar. Hosts and symbionts were then kept together in a 300 L open-water tank with a sandy substrate for a minimum of 24 h until further experimentation. During that time, the symbionts could switch from one host individual to another. Prior to the behavioural experiments, the seastars and shrimps were separated and isolated individually in 30 L tanks for a minimum of three hours. All behavioural experiments were performed using 1 µm-filtered seawater, with a temperature of 28–29 °C and a salinity of 35‰, pumped directly from the Opunohu Bay facing the marine station. For asterosaponin extractions (see here below), five *C*. *novaeguineae* and one crown-of-thorns seastar *Acanthaster* *planci* (Linnaeus, 1758) were harvested. The latter was hand-collected by scuba diving at 20 m depth, on the external reef outside the Opunohu Bay (17° 28′ 51″ S; 149° 51′ 21″ W; Fig. 1A,B).

To obtain *Z.* *soror* larvae (Fig. 1F), 15 gravid shrimps were kept in low-volume individual containers under a constant light and moderate elevated temperature (29 °C) for 6 h at different periods of the day. The eggs of five females hatched around 10 PM and the larvae were used directly for behavioural experiments. During these treatments, eggs were regularly collected on the bottom of the container and characterized under binoculars to evaluate the embryonic development of the egg. For each developmental stage investigated, n = 6 propagules and n = 10 zoeal larvae were considered for the description.

After experimentation, all cushion seastars and their associated shrimps were returned to their original location in the lagoon, with the exception of those preserved for saponin extractions. Animals used for experiments were maintained and treated in compliance with the guidelines specified by the French Polynesia’s Environment Department (DIREN) and by the Belgian Ministry of Trade and Agriculture.

### Chemical characterization

The behavioural experiments on adult shrimps and their larvae (see here below) required the chemical extraction of asterosaponins from three hosts and three non-host species. The non-host species, *Echinaster* *sepositus*, *Asterias* *rubens*, and *Holothuria* *scabra* were not collected in Mo’orea but in the Mediterranean Sea (Algiers bay; Pointe Pescade; Algeria) and North Atlantic Ocean (Audresselles; Opal Coast; France) for the first two seastars, and the Mozambican channel (Great Reef of Toliara; Madagascar) for the third one, a sea cucumber. They have not been previously recorded as associates of *Z.* *soror* which is exclusively associated with tropical seastars^12,32–36^. The contents of saponins in these species have previously been thoroughly characterized in our laboratory^30,54,55^ and preserved chemical extracts from these studies were used for olfactometer experiments. The extraction and mass spectrometry analyses of asterosaponins from the potential host species, *C.* *novaeguineae*, *A.* *planci*, collected in Mo’orea, and *Linckia* *laevigata*, from the Great Reef of Toliara (Madagascar) were conducted using the following standardized methodology.

### Saponin extractions

The body walls of each species were dissected and lyophilized with an Alpha 1–2 freeze dryer for 48 h to preserve the chemical nature of the metabolites. The dried samples were then finely ground using a mechanical crusher IKA A11 and homogenized in 100% methanol technical grade solution for 12 h. The methanolic extract was recovered through centrifugation (10 min; 4500 rpm) and diluted with Milli-Q water to reach a 70:30 MeOH:H_2_O ratio. It was partitioned successively through three liquid–liquid extractions using 100% *n*-hexane C_6_H_14_ technical grade (*v*:*v*), 100% chloroform CHCl_3_ technical grade (*v*:*v*) and finally 100% dichloromethane CH_2_Cl_2_ technical grade (*v*:*v*). The final hydromethanolic solution was evaporated using a rotary evaporotator (Laborata 4001 efficient, Heidolph) at low pressure in a double boiler at 60/65 °C. The dry residue was redissolved in Milli-Q water and was then purified using the last liquid/liquid extraction with isobutanol HPLC grade (*v*:*v*). The solution was washed three times with Milli-Q water to remove inorganic salts and recover the purified saponins, and this butanolic fraction, containing the dissolved saponins, was re-dried using the rotary evaporator with the same parameters described earlier. The dry extracts were weighed and stored in the dark at − 20 °C until use in olfactometry experiments or mass spectrometry analyses.

### Mass spectrometry analyses

The purified asterosaponin extracts underwent two types of spectrometric analyses^71^: (1) identification and quantification of the saponins present in the extracts and (2) exact mass analysis. All the mass spectra were obtained using a mass spectrometer (Waters SYNAPT G2-S*i*) coupled with a liquid chromatography device (Waters Acquity H-class) and analysed with MassLynx 4.1 software (Waters).

Liquid chromatography was performed with 5 μl of the sample on an ACQUITY UPLC BEH C18 reverse phase UPLC column, 130 Å, 1.7 μm, 2.1 mm × 50 mm at 40 °C with a gradient of eluent A, consisting of a 60/40 mixture of a solution of milli-Q water and formic acid HPLC grade (HCOOH, 0.1%) and eluant B composed of acetonitrile HPLC grade at a constant rate of 250 μl/min as explained in Brasseur et al.^22^. Considering that the vast majority of currently described asterosaponins in seastars have a sulphate moiety^30,58–60^, mass spectrometry analyses were performed via electrospray negative (−) or positive (+) ionization of the molecules (ESI). Note that, in the present study, we did not intend to structurally characterize all the saponin molecules which usually requires other techniques such as proton high magnetic resonance. We thus only reported the saponin elemental compositions with regards to the LC elution times, see Table 1.

The following parameters were used for the ESI: a capillary voltage of 2.5 kV (−) or 3.1 kV (+), a cone voltage of 40 V, a source offset of 80 V, a source temperature of 100 °C and a desolvation temperature of 300 °C. The ESI gas used was dry nitrogen supplied at a flow rate of 60 L/h for the gas cone and 500 L/h for the desolvation gas. The quadrupole was set to pass ions from *m/z* 50 to 2000, all the ions were transmitted to the pusher region of the time-of-flight analyser with an integration time of 1 s. A quantitative comparison was made for each congener by comparing the area under their LC spectrum peaks to an internal reference, hederacoside C, which also allowed determining the sample purity. To obtain the exact masses of saponin ions, the analyses were carried out in positive mode via electrospray ionization (ESI) of the molecules with the Hederacoside C ions ([M−H]^−^—*m/z* 1219.6, [M+H]^+^—*m/z* 1221.6) as the internal standard (lock mass).

The relative quantification of natural extracts was achieved by adding a known quantity (0.1 mg mL^−1^) of commercially available hederacoside C (Sigma-Aldrich-Product n° 97151-M-ClarityTM Program MQ100), as an internal standard in all solutions of saponin extract at a given concentration, typically 0.1 mg·mL^−1^. The spiked solution was analysed using LC–MS (injection of 5 µL) using the experimental conditions described here above. For each saponin molecule, including hederacoside C, the corresponding LC–MS ion signals—including all the isotopic compositions—were integrated using the integration algorithm available under MassLynxTM 4.1 Software. The global ion counts were then used to estimate the relative concentration (mol-%) of each saponin congener within the saponin cocktail. The %-weights in extract correspond to the mass percentages of saponin congeners relative to hederacoside C within the saponin extracts. Note that the sum of the %-weight does not reach 100% allowing to estimate the saponin content (purity) within the extract.

### Behavioural experiments

**Figure 6:**
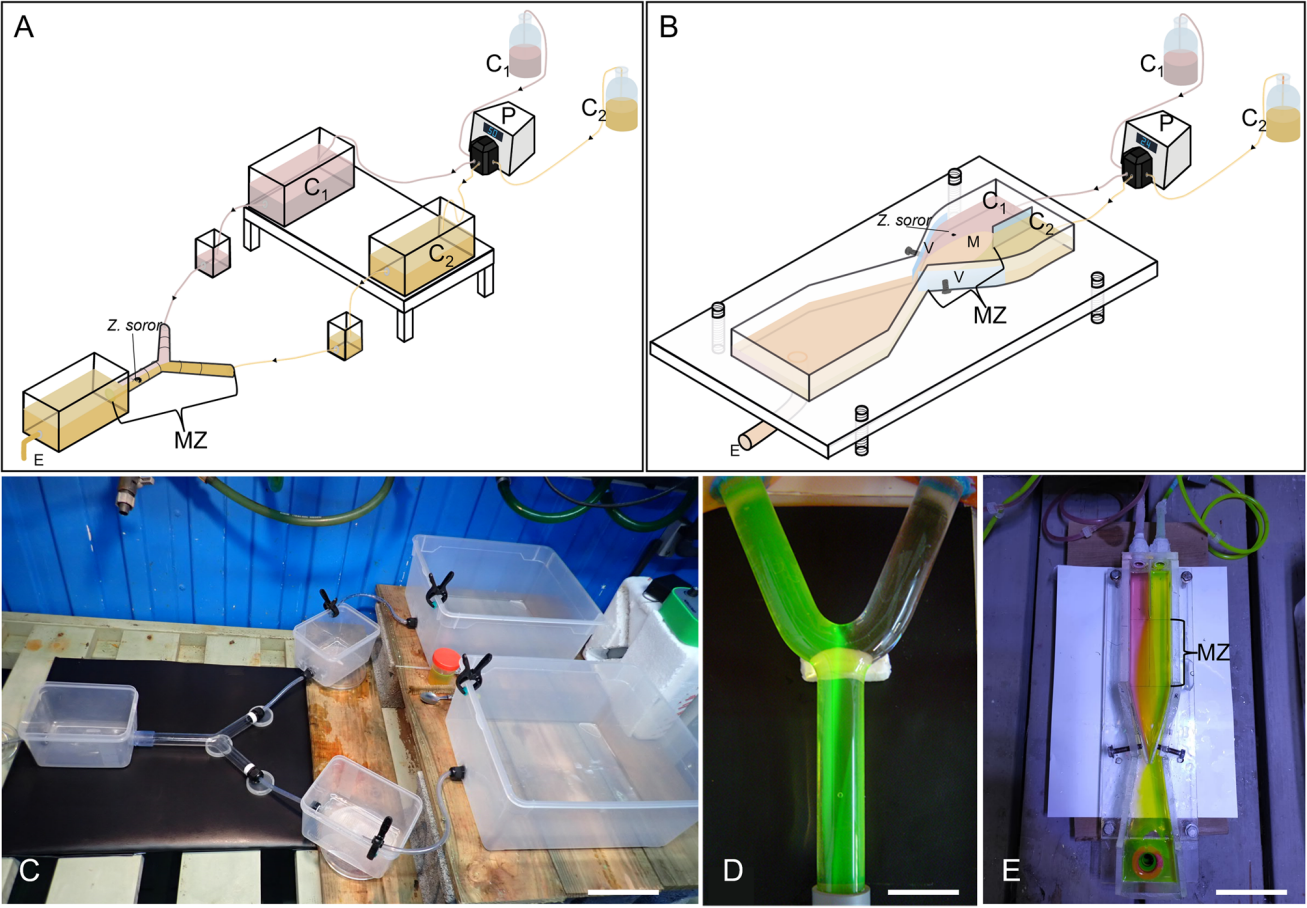
Experimental devices used in behavioural experimentations: (**A**) olfactometer used for adults *Z.* *soror*. (**B**) low-flow olfactometer used for *Z.* *soror* larvae. *C1* chemical cue 1, *C2* chemical cue 2, *M* mix of both cues, *MZ* monitoring zone, *P* peristaltic pump, *V* valve composing the valve regulation system, *E* exit. (**C**) Real experimental device used for adult *Z.* *soror*. (**D**) Zoom of the Y-tube (corresponding to the monitoring zone) used for adult *Z.* *soror*, with the visualization of the flow laminarity using fluorescein and (**E**) real low-flow olfactometer used for *Z.* *soror* larvae, with the visualization of the flow laminarity using food colourants. *MZ* = monitoring zone. Scale bars represent 13.3 cm in (**C**); 3.3 cm in (**D**); 5 cm in (**E**).

The chemical communication behaviour between the seastar shrimps and the different host and non-host species was studied using two types of olfactometers (Fig. 6) including an innovative Y-tube device made-to-measure larval chemotaxis under low flow condition (Fig. 6B,E). The experiments were conducted in two stages: (1) investigation of the chemical communication between adult and larvae of *Z.* *soror* and their host species *C.* *novaeguineae*, and (2) identification of the potential kairomonal role of saponins extracted from various species. One of the aquaria (A) of the olfactometer contained conditioned seawater with either living seastars or purified saponins, while the other (B) was filled with control seawater. Test water consisted of seawater conditioned either with living seastars (i.e. conditioned with 2 individuals in a 10 L-aquarium for 2 h) or with purified asterosaponins from *C.* *novaeguineae* or *A.* *planci* diluted with a concentration of 0.01 mg L^−1^ (determined empirically in preliminary assays). Flow laminarity, flow speed inside the olfactometers was controlled using fluorescein salt before the experiments (Fig. 6D,E). A minimum of 20 replicates using 20 adult shrimps (using the three different morphotypes) were performed for each experiment, and the aquaria were exchanged every 10 runs (to consider any variations that could exist on either side of the olfactometers). Once tested, each adult shrimp individual was left on their host for 10 days between experiments. A minimum of twenty larvae were also used in one experiment (20 assays) and each larva was tested only once directly after hatching. The entire olfactometer was washed after each trial. A negative control, with both aquaria filled with seawater, was performed on experiments using adults and larvae. Experiments were performed in a dark room with artificial low-intensity LED lights.

### Olfactometer experiments on *Z.soror* adults

The chemical attraction of *Z.* *soror* adults towards *C.* *novaeguineae* was evaluated using a Y-tube olfactometer system (Fig. 6A,C,D) similar to the one described in several previous studies^13,21,22^. The system consisted of a 20 cm long unpaired branch connected to two 10 cm paired branches that were each connected to a 10 L-aquarium. The glass tube diameter was 3 cm. Water flowed from these two aquaria through the olfactometer and was evacuated at the base of the unpaired branch of the Y-tube where the shrimp was introduced at the beginning of each test and the water flow was regulated to a speed of 2–3 cm s^−1^. An attractive chemical communication was recorded when a significant proportion of shrimps moved up the flow and orientated towards the olfactory stimulus source. During a typical trial, one *Z.* *soror* was introduced at the base of the unpaired branch of the Y tube. If it was not stimulated, the shrimp remained at the base of the branch without moving and the run was aborted and considered as null after 5 min (null). If the shrimp was stimulated, it moved into the unpaired branch up to the junction of the two-paired branches where it usually stopped moving, tested the water fluxes, and entered one of the paired branches. Two types of behaviours were therefore recorded in the Y tube: the motion behaviour, when shrimps moved for at least 10 cm into the unpaired branch towards the stimulation source, and the orientation behaviour, when shrimps entered one of the two paired branches and reached the corresponding aquarium ^13,15,16^. Videos SI1 and SI2 illustrate the motion and the orientation behaviours of the shrimp *Z.* *soror* in Y-tubes. Video SI1 represents a typical *Z.* *soror* that behaves in response to a positive chemical stimulus (positive motion and orientation behaviour) and video si2 corresponds to a shrimp that is not attracted by any chemical cue and remains still in the device (null result). Those videos were recorded for illustration only as typical tests were not filmed to avoid any bias and were performed in low light condition.

### Olfactometer experiments on *Z.soror* larvae

The chemical attraction of *Z.* *soror* larvae towards *C.* *novaeguineae* was evaluated in a new innovative low-flow olfactometer device (Fig. 6B,E) designed to measure chemotaxis in a low current allowing the larvae to swim in the system. It consisted of a 25 cm long plexiglass compartment, with the upper extremity connected to a dual flow peristaltic Cole-Parmer MasterFlex^®^ pump bringing cues from two 10 L-aquaria. Water flowed from the upper extremities through the olfactometer and was evacuated at the base of the bottom extremity. The two flows were kept separated by a separation thin wall on the first 3 cm of the device, to stabilize and discriminate the two laminar flows. Downstream in the system, the flows mixed creating a chemical gradient (Fig. 6B,E). The monitoring zone (i.e. where the larvae were introduced and their behaviours were recorded) was comprised between the boundary where cues mix together and the valve regulation system limiting the flow speed. Flow laminarity inside the olfactometer was controlled using two different food colouring before experiments (Fig. 6E) and the water current was regulated with a pump to 24 mL min^−1^. An attractive chemical communication was recorded when a significant proportion of larvae swam actively and orientated towards the olfactory stimulus source. If it was not stimulated, the larvae remained immobile and were passively transported (i.e. drifting) through the monitoring area without a clear orientation in less than 30 s. In that case, the trial was considered as Null. During a typical trial, if larvae were stimulated, they moved actively and could choose a side of the device while actively progressing against the low current. Two types of behaviours were therefore recorded in larvae olfactometers: the motion behaviour corresponding to the observation of swimming movements of the larvae in the monitoring zone and the orientation behaviour that was measured with the position record occurring every 5 s (left, middle or right in the monitoring zone) of the device. The drifting time the larvae that stayed inside the monitoring zone were also recorded, with a maximum of 200 s that corresponded to the end of the experimentation.

### Statistical analyses

The motion behaviour of adult shrimps and larvae was evaluated by comparing the number of times they started to move under cue stimulation (chemotaxis) to the number of times they moved in response to control seawater (negative test), using a Pearson's chi-squared test with Yates continuity correction (χ^2^ = 0.05, df = 1). The orientation behaviour of adult shrimps and larvae was determined by comparing the percentage of individuals that oriented towards the side with a chemical to a random distribution (binomial test, probability of success = 0.5). The duration that larvae remained in the low-flow device was assessed using an analysis of variance (ANOVA) considering a significant drifting time difference between tests at the α level of 5%. Multiple comparisons of means (Tukey HSD) were performed to compare the results. All statistical tests were performed with the R software (R project, 0.64 3.2.3; R Core Team). For every test, results were considered significant when the P value is legal or inferior to 0.01.

### Approval for animal experiments

The sampling and experimentation were made according to the permit and APA norms provide by the “Direction de l’Environnement de Polynésie française” (DIREN).

### Supplementary Information

Supplementary Information.

## Data Availability

The data presented in this study are available in the supplementary material and are available on request from the A.L. or G.C.

## References

[1] Paracer S, Ahmadjian V (2000). Symbiosis: An Introduction to Biological Associations.

[2] Parmentier E, Michel L (2013). Boundary lines in symbiosis forms. Symbiosis.

[3] Trilles J-P, Hipeau-Jacquotte R (2012). Treatise on Zoology—Anatomy, Taxonomy, Biology. The Crustacea.

[4] Johnstone RA, Bshary R (2002). From parasitism to mutualism: Partner control in asymmetric interactions. Ecol. Lett..

[5] González R (2021). Plant virus evolution under strong drought conditions results in a transition from parasitism to mutualism | PNAS. Proc. Natl. Acad. Sci. USA.

[6] Lanterbecq D, Rouse GW, Eeckhaut I (2010). Evidence for cospeciation events in the host–symbiont system involving crinoids (Echinodermata) and their obligate associates, the myzostomids (Myzostomida, Annelida). Mol. Phylogenet. Evol..

[7] Horká I, De Grave S, Fransen CHJM, Petrusek A, Ďuriš Z (2016). Multiple host switching events shape the evolution of symbiotic palaemonid shrimps (Crustacea: Decapoda). Sci. Rep..

[8] Gherardi F (1991). Eco-ethological aspects of the symbiosis between the shrimp *Athanas*
*indicus* (Coutière 1903) and the sea urchin *Echinometra*
*mathaei* (de Blainville 1825). Trop. Zool..

[9] Eeckhaut I, Jangoux M (1997). Infestation, population dynamics, growth and reproductive cycle of *Myzostoma*
*cirriferum* (Myzostomida), an obligate symbiont of the comatulid crinoid *Antedon*
*bifida* (Crinoidea, Echinodermata). Cah. Biol. Mar..

[10] Derby CD, Sorensen PW (2008). Neural processing, perception, and behavioral responses to natural chemical stimuli by fish and crustaceans. J. Chem. Ecol..

[11] Ache BW, Davenport D (1972). The sensory basis of host recognition by symbiotic shrimps, genus *Betaeus*. Biol. Bull..

[12] Antokhina TI, Britayev TA (2020). Host recognition behaviour and its specificity in pontoniine shrimp* Zenopontonia soror* (Nobili, 1904) (Decapoda: Caridea: Palaemonidae) associated with shallow-water sea stars. J. Exp. Mar. Biol. Ecol..

[13] Caulier G, Flammang P, Gerbaux P, Eeckhaut I (2013). When a repellent becomes an attractant: Harmful saponins are kairomones attracting the symbiotic Harlequin crab. Sci. Rep..

[14] Eeckhaut I, VandenSpiegel D, Michel A, Jangoux M (2000). Host chemodetection by the crinoid associate *Harrovia*
*longipes* (Crustacea: Brachyura: Eumedonidae) and a physical characterization of a crinoid-released attractant. Asian Mar. Biol..

[15] Fourgon D, Jangoux M, Eeckhaut I (2007). Biology of a “babysitting” symbiosis in brittle stars: Analysis of the interactions between *Ophiomastix*
*venosa* and *Ophiocoma*
*scolopendrina*. Invertebr. Biol..

[16] Dicke M, Sabelis MW (1988). Infochemical terminology: Based on cost-benefit analysis rather than origin of compounds?. Funct. Ecol..

[17] Dimock RV, Davenport D (1971). Behavioral specificity and the induction of host recognition in a symbiotic polychete. Biol. Bull..

[18] Gage J (1966). Experiments with the behaviour of the bivalves *Montacuta*
*substriata* and *M.*
*ferruginosa*, ‘commensals’ with spatangoids. J. Mar. Biol. Assoc. UK.

[19] Zimmer R, Butman C (2000). Chemical signaling processes in the marine environment. Biol. Bull..

[20] Murata M, Miyagawa-Kohshima K, Nakanishi K, Naya Y (1986). Characterization of compounds that induce symbiosis between sea anemone and anemone fish. Science.

[21] Caulier G (2022). Crinoid anthraquinones as kairomones allowing host selection for the symbiotic snapping shrimp *Synalpheus*
*stimpsonii*. Chemoecology.

[22] Brasseur L, Caulier G, Lepoint G, Gerbaux P, Eeckhaut I (2018). *Echinometra*
*mathaei* and its ectocommensal shrimps: The role of sea urchin spinochrome pigments in the symbiotic association. Sci. Rep..

[23] Metcalf RL, Metcalf ER (1992). Plant Kairomones in Insect Ecology and Control.

[24] Vance RR (1973). On reproductive strategies in marine benthic invertebrates. Am. Nat..

[25] Hadfield MG, Paul VJ (2001). Natural chemical cues for settlement and metamorphosis of marine invertebrate larvae. Mar. Chem. Ecol..

[26] Lecchini D (2017). Habitat selection by marine larvae in changing chemical environments. Mar. Pollut. Bull..

[27] Doll PC (2023). Settlement cue selectivity by larvae of the destructive crown-of-thorns starfish. Biol. Lett..

[28] Cowan Z-L, Dworjanyn SA, Caballes CF, Pratchett M (2016). Benthic predators influence microhabitat preferences and settlement success of crown-of-thorns starfish (*Acanthaster*
*cf.*
*solaris*). Diversity.

[29] Marsden JR (1987). Coral preference behaviour by planktotrophic larvae of *Spirobranchus*
*giganteus*
*corniculatus* (Serpulidae: Polychaeta). Coral Reefs.

[30] Demeyer M (2014). Molecular diversity and body distribution of saponins in the sea star *Asterias*
*rubens* by mass spectrometry. Comp. Biochem. Physiol. B Biochem. Mol. Biol..

[31] Claereboudt EJS (2023). A distinct saponin profile drives an olfactory-mediated aggregation in the aquacultivated sea cucumber *Holothuria*
*scabra*. Mar. Drugs.

[32] Schelvis M (2017). The association between the sea star shrimp *Periclimenes*
*soror* and its asteroid hosts: Assessing preferences in host species and food choice. Biology.

[33] Bruce AJ (1976). *Periclimenes*
*soror* Nobili, a Pontoniin shrimp new to the american fauna, with observations on its indo-west pacific distribution. Tethys.

[34] Olliff E (2013). Symbiosis of the sea star shrimp, *Periclimenes*
*soror* Nobili, 1904 (Decapoda, Palaemonidae), and cushion star, *Culcita*
*novaeguineae* Müller & Troschel, 1842 (Echinodermata, Asteroidea, Oreasteridae): Host fidelity, host finding and benefits. Crustaceana.

[35] Guenther J, Walker-Smith G, Warén A, De Nys R (2007). Fouling-resistant surfaces of tropical sea stars. Biofouling.

[36] Sakachi H, Okutani T (1988). On the relationship between the starfish and the starfish Perichimenes soror NOBILI in Kuroshima, Yaeyama Islands. Crustacean Studies.

[37] Correa C, Thiel M (2003). Mating systems in caridean shrimp (Decapoda: Caridea) and their evolutionary consequences for sexual dimorphism and reproductive biology. Rev. Chil. Hist. Nat..

[38] Li X (2008). Report on some species of Palaemonidae (Crustacea, Decapoda) from French Polynesia. Zoosystema.

[39] Poupin J (1998). Crustacea decapoda and stromatopoda of French polynesia. Atoll Res. Bull..

[40] Clark C, Weitzman B (2008). Population Study Survey of Acanthaster planci, the Crown-of-Thorns Starfish on the North-West Coast Moorea.

[41] Krupa J, Reeves C (2004). Acanthaster planci Population Survey on the North Coast of Moorea.

[42] Kayal M, Kayal E (2017). Colonies of the fire coral *Millepora*
*platyphylla* constitute scleractinian survival oases during *Acanthaster* outbreaks in French Polynesia. Mar. Biodiv..

[43] Adjeroud M, Salvat B (1996). Spatial patterns in biodiversity of a fringing reef community along Opunohu Bay, Moorea, French Polynesia. Bull. Mar. Sci..

[44] Antokhina TI, Britayev TA (2012). Sea stars and their macrosymbionts in the Bay Of Nhatrang, Southern Vietnam. Paleontol. J..

[45] Tseng L-C, Limviriyakul P, Hwang J-S (2022). Macrosymbionts of starfish *Echinaster*
*luzonicus* (Gray, 1840) in the waters of a volcanic western Pacific island. PLoS One.

[46] Caulier G, Parmentier E, Lepoint G, Neder FV, Eeckhaut I (2012). Characterization of a population of the Harlequin crab, *Lissocarcinus*
*orbicularis* Dana, 1852, an obligate symbiont of holothuroids, in Toliara bay (Madagascar). Zoosymposia.

[47] Nazari EM, Simões-Costa MS, Müller YMR, Ammar D, Dias M (2003). Comparisons of fecundity, egg size, and egg mass volume of the freshwater prawns *Macrobrachium*
*potiuna* and *Macrobrachium*
*olfersi* (Decapoda, Palaemonidae). J. Crustac. Biol..

[48] Cuvin-Aralar MLA (2014). Embryonic development of the Caridean prawn *Macrobrachium*
*mammillodactylus* (Crustacea: Decapoda: Palaemonidae). Invertebr. Reprod. Dev..

[49] Wowor D (2009). Evolution of life history traits in Asian freshwater prawns of the genus *Macrobrachium* (Crustacea: Decapoda: Palaemonidae) based on multilocus molecular phylogenetic analysis. Mol. Phylogenet. Evol..

[50] Little G (1968). Induced winter breeding and larval development in the shrimp, *Palaemonetes*
*pugio* Holthuis (Caridea, Palaemonidae). Crustaceana.

[51] Wear RG (1976). Larva of the commensal shrimp *Periclimenes*
*(Periclimenes)*
*soror* Nobili, 1904 (Crustacea: Decapoda: Pontoniinae) from Fiji. NZ J. Mar. Freshw. Res..

[52] Antokhina TI, Sorokin PA (2010). Molecular genetic analysis of the two morphs of sea star shrimp *Periclimemes*
*soror* Nobili, 1904, the symbionts of tropic sea stars. Genetika.

[53] Motti CA (2018). Chemical ecology of chemosensation in asteroidea: Insights towards management strategies of pest species. J. Chem. Ecol..

[54] Dahmoune B (2021). Saponin contents in the starfish *Echinaster*
*sepositus*: Chemical characterization, qualitative and quantitative distribution. Biochem. Syst. Ecol..

[55] Savarino P (2022). Microwave-assisted desulfation of the hemolytic saponins extracted from *Holothuria*
*scabra* Viscera. Molecules.

[56] Immelmann K (1975). Ecological significance of imprinting and early learning. Annu. Rev. Ecol. Syst..

[57] VandenSpiegel D, Eeckhaut I, Jangoux M (1998). Host selection by *Synalpheus*
*stimpsoni* (De Man), an ectosymbiotic shrimp of comatulid crinoids, inferred by a field survey and laboratory experiments. J. Exp. Mar. Biol. Ecol..

[58] Kicha AA (2014). Minor steroidal triglycoside planciside D from the tropical starfish *Acanthaster*
*planci*. Chem. Nat. Compd..

[59] Ha DT (2021). Asterosaponins from the tropical starfish *Acanthaster*
*planci* and their cytotoxic and anticancer activities in vitro. Nat. Prod. Res..

[60] Stonik VA, Kicha AA, Malyarenko TV, Ivanchina NV (2020). Asterosaponins: Structures, taxonomic distribution, biogenesis and biological activities. Mar. Drugs.

[61] Ngoan BT (2015). Asterosaponins and glycosylated polyhydroxysteroids from the starfish *Culcita*
*novaeguineae* and their cytotoxic activities. J. Asian Nat. Prod. Res..

[62] Kasumyan A (2020). Comatulids (Crinoidea, Comatulida) chemically defend against coral fish by themselves, without assistance from their symbionts. Sci. Rep..

[63] Tang H-F (2005). Bioactive Asterosaponins from the Starfish *Culcita*
*novaeguineae*. J. Nat. Prod..

[64] Lu Y (2018). Cytotoxic polyhydroxysteroidal glycosides from starfish *Culcita*
*novaeguineae*. Mar. Drugs.

[65] Ma XG (2009). Two new 24-hydroxylated asterosaponins from *Culcita*
*novaeguineae*. Chin. Chem. Lett..

[66] Lu Y-Y (2022). Two new polyhydroxylated steroidal glycosides from the starfish *Culcita*
*novaeguineae*. Nat. Prod. Res..

[67] Lu Y-Y (2020). Chemical constituents from starfish *Culcita*
*novaeguineae*. Biochem. Syst. Ecol..

[68] Iorizzi M, Minale L, Riccio R, Higa T, Tanaka J (1991). Starfish saponins, Part 46. Steroidal glycosides and polyhydroxysteroids from the starfish *Culcita*
*novaeguineae*. J. Nat. Prod..

[69] Levina EV, Kalinovsky AI, Dmitrenok PS, Martyyas EA, Stonik VA (2010). Two new steroidal Saponins, Hylodoside A and Novaeguinoside Y, from the Starfish *Leptasterias*
*hylodes*
*reticulata* and *Culcita*
*novaeguineae* (Juvenile). Nat. Prod. Commun..

[70] Kitagawa I, Kobayashi M (1977). On the structure of the major saponin from the starfish acanthaster planci. Tetrahedron Lett..

[71] Savarino P, Demeyer M, Decroo C, Colson E, Gerbaux P (2021). Mass spectrometry analysis of saponins. Mass Spectrom. Rev..

