# Supplementary material for: Species-specific metabolites mediate host selection and larval recruitment of the symbiotic seastar shrimp
Source: Sci Rep. 2023 Aug 4;13:12674. doi: 10.1038/s41598-023-39527-2 (PMC10403617; doi:10.1038/s41598-023-39527-2)
Supplement: Supplementary file 1 — Supplementary Information. [file 41598_2023_39527_MOESM1_ESM.pdf]

Supplementary Material

Species-specific metabolites mediate host selection and larval recruitment of the symbiotic seastar shrimp

Alexia Lourtie[+ 1,2]; Igor Eeckhaut[1,3*]; Jérôme Mallefet[2*]; Philippe Savarino[4]; Mathilde Isorez[1]; Lisa Mussoi[1]; Hugo Bischoff[5,6]; Jérôme Delroisse[1]; Laetitia Hédouin[5,6]; Pascal Gerbaux[4]; Guillaume Caulier[+ 1,3]

[1] University of Mons - UMONS, Research Institute for Biosciences, Biology of Marine Organisms and Biomimetics Unit. 23 Place du Parc, B-7000 Mons, Belgium.

[2] University UCLouvain, Earth and Life Institute, Marine Biology Laboratory, Croix du sud 3/L7.06.04, B-1348 Louvain-la-Neuve, Belgium.

[3] Belaza Marine Station (IH.SM-UMONS-ULIEGE), Toliara, Madagascar.

[4] University of Mons - UMONS, Research Institute for Biosciences, Organic Synthesis and Mass Spectrometry Laboratory. 23 Place du Parc, B-7000 Mons, Belgium.

[5] PSL Research University: EPHE-CNRS-UPVD, USR 3278 CRIOBE, BP 1013, 98729, Papetoai, Mo'orea, French Polynesia.

[6] Laboratoire D'Excellence CORAIL, Mo'orea, French Polynesia.

[+] *Corresponding authors: alexia.lourtie@umons.ac.be ; guillaume.caulier@umons.ac.be; +32476226822; ORCID: 0000-0002-8227-670X*

[*] These authors contributed equally to the realization of this article and should be considered as co-second authors.

Supplementary material (Tables, figures, data)

Supplementary Material

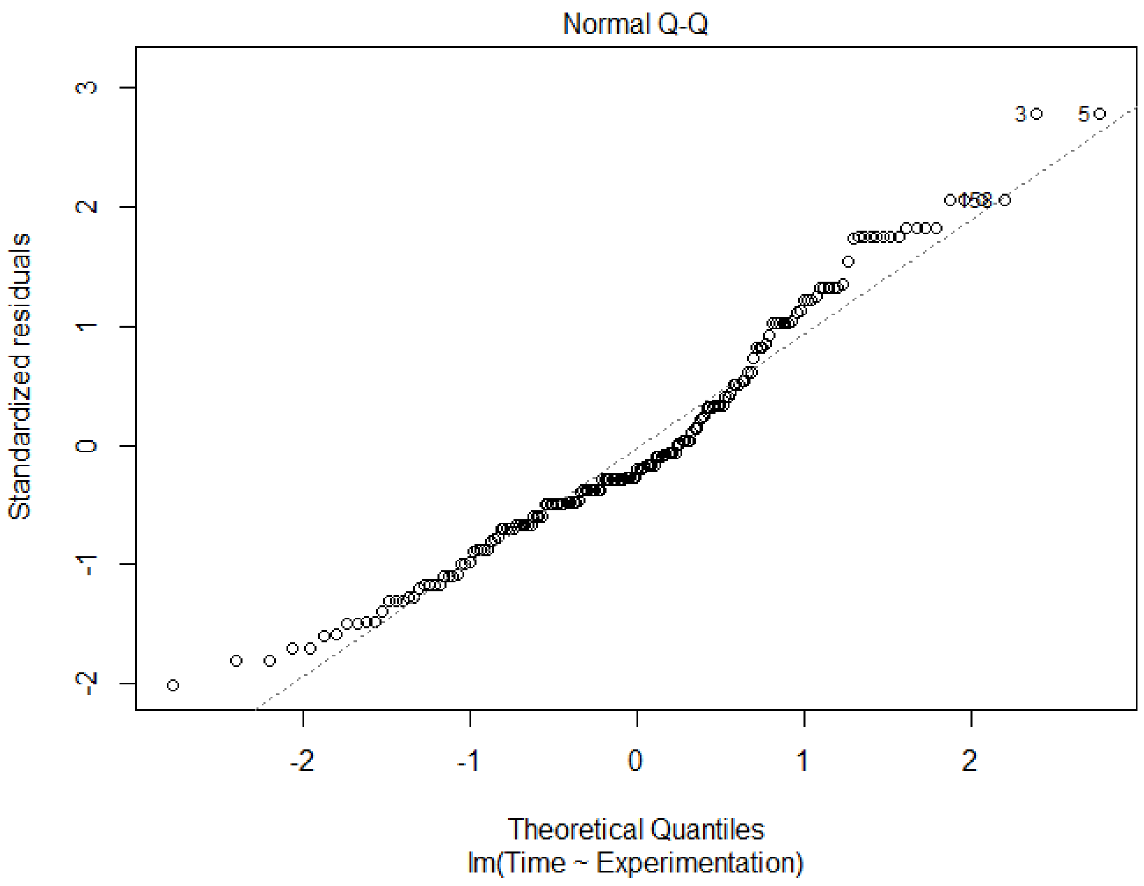

Figure SI-1 : **Quantile - Quantile (Q-Q) plot:** represent the distribution of the residuals compared to a normal distribution prior the realization of the ANOVA analysis (see **Fig. 4**) comparing the mean deriving times between experimentations. X-axis = theoretical quantiles of the standard normal distribution ; Y-axis = quantiles of the residuals. The points on the plot represent the residuals from the ANOVA model. The diagonal line on the plot represents the line of equality, where the theoretical quantiles are equal to the measured quantiles. Points that correspond to this theoretical line signifies that the residuals are normally distributed. Deviations from the diagonal line indicate departures from normality.

Supplementary Material

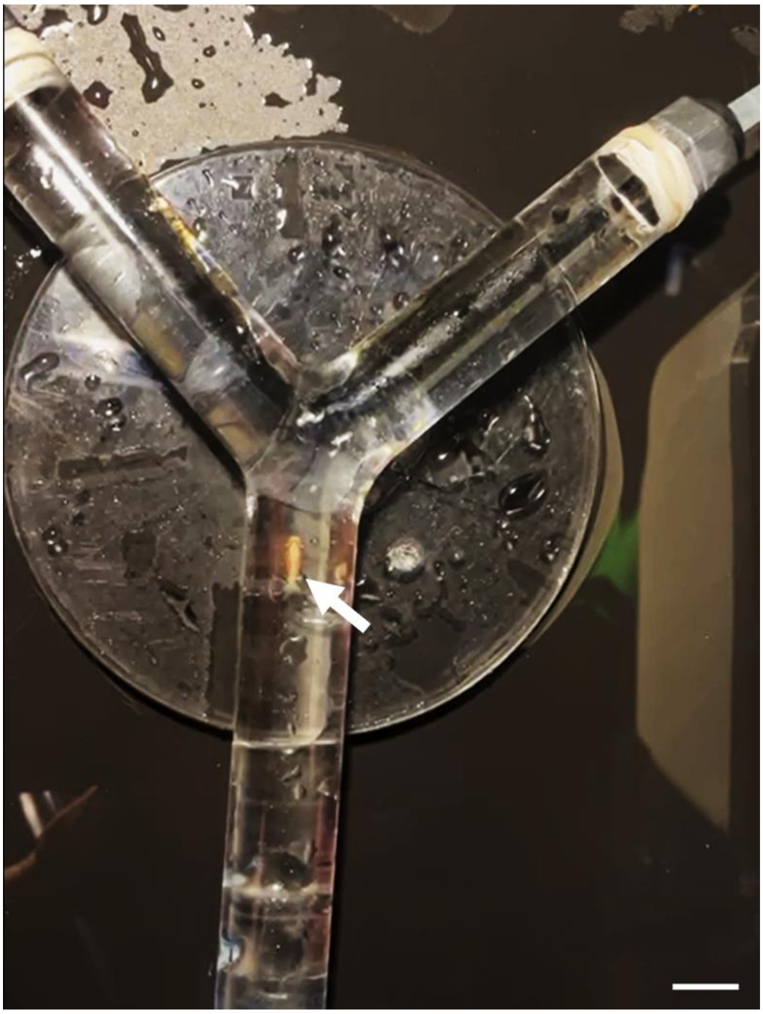

Video SI-1 :Positive motion and orientation behaviour of *Zenopontonia soror*: https://youtube.com/shorts/5SONg0VJaZs ; video illustrating the motion and orientation behaviour performed by an asteroid shrimp *Z. soror* (highlighted by a white arrow) into a Y-tube olfactometer. The scale bar represents 2 cm.

Supplementary Material

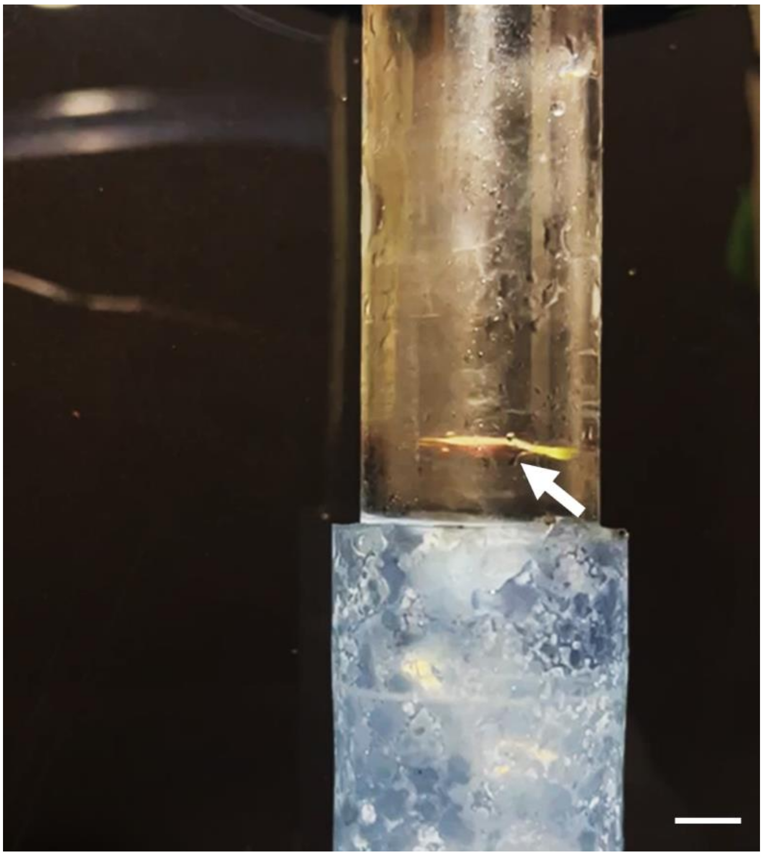

Video SI-2 : **Absence of motion behaviour of *Zenopontonia soror:*** https://youtube.com/shorts/S0wDFKK81po ; video illustrating the absence of motion and orientation behaviour (null result) performed by an asteroid shrimp *Z. soror* (highlighted by a white arrow) into a Y-tube olfactometer. The scale bar represents 0.8 cm.
